# *Panax notoginseng* preparations as adjuvant therapy for diabetic kidney disease: a systematic review and meta-analysis

**DOI:** 10.1080/13880209.2020.1711782

**Published:** 2020-01-22

**Authors:** Xiuge Tang, Mingdi Huang, Junsong Jiang, Xueyan Liang, Xianshu Li, Ruqing Meng, Lingyuan Chen, Yan Li

**Affiliations:** a Department of Cardiovascular Medicine, The People’s Hospital of Hechi, Hechi, PR China; b Department of Nephrology, The People’s Hospital of Hechi, Hechi, PR China; c Department of Reproductive Medicine, The People’s Hospital of Hechi, Hechi, PR China; d Department of Pharmacy, The People’s Hospital of Hechi, Hechi, PR China

**Keywords:** *Panax notoginseng* saponins, kidney function, serum lipid, fasting blood glucose

## Abstract

**Context:**

*Panax notoginseng* (Burk.) F.H. Chen (Araliaceae) preparations (PNP) are traditional Chinese medicines used as adjuvant therapeutics for diabetic kidney disease (DKD).

**Objective:**

To systematically review the efficacy of PNP as adjunct DKD therapy, including their effects on kidney function, serum lipid levels and fasting blood glucose levels.

**Methods:**

The databases PubMed, Embase, Medline, Cochrane Library, CINAHL, China Biology Medicine disc, Wanfang, VIP and China National Knowledge Infrastructure were systematically searched from the date of their inception until May 2019. *Panax notoginseng*, *Panax notoginseng* saponins, Lulutong, Xueshuantong and Xuesaitong were the key terms searched. Randomized controlled trials (RCTs) comparing the combined use of PNP and conventional medicines (CM) versus CM for DKD were included. Data were pooled using random or fixed effect models depending on heterogeneity.

**Results:**

In total, 24 RCTs involving 1918 participants were analysed. Adjunct PNP with CM was associated with reduction of albuminuria (MD –26.89 mg, 95% CI: –33.35 to –20.42), proteinuria (MD –0.32 g/24 h, 95% CI: –0.36 to –0.27), serum creatinine (MD –4.52 μmol/L, 95% CI: –8.71 to –0.32), total cholesterol (MD –1.56 mmol/L, 95% CI: –2.33 to –0.78), triglycerides (TG) (MD –0.56 mmol/L, 95% CI: –0.80 to –0.31) and low-density lipoprotein cholesterol (MD –0.94 mmol/L, 95% CI: –1.49 to –0.40) compared with CM.

**Conclusions:**

This is the first meta-analysis investigating adjuvant PNP therapy for DKD. PNP apparently exerted beneficial effects on kidney function and improved the metabolism of serum lipids by CM. Further, well-conducted, high-quality trials on DKD patients are needed to provide high-quality evidence.

## Introduction

Diabetic kidney disease (DKD) is a serious complication of diabetes mellitus that is widespread globally. Approximately, 35–40% of patients with type 1 or 2 diabetes eventually develop DKD, which significantly increases their mortality rate and poses a serious threat for their clinical outcomes (Yang et al. 2018). According to projected data from the International Diabetes Federation (IDF), the number of diabetic patients worldwide will increase from 382 million in 2013 to 592 million by 2035 (Shi and Hu 2014). DKD is one of the most significant risk factors for end-stage kidney disease (ESKD) and requires long-term dialysis or even a kidney transplant (Tuttle et al. 2014; Alicic et al. 2017; Doshi and Friedman 2017). Kidney disease attributed to diabetes is a major contributor to the global burden of the disease (Alicic et al. 2017); furthermore, both the social economic and public health burden of DKD are significant (Sarnak et al. 2003; Atkins and Zimmet 2010; Radcliffe et al. 2017). Contemporary treatment options and promising new therapies for DKD are of critical importance.

Two of the most prominent established DKD risk factors are hyperglycaemia and hypertension (Alicic et al. 2017). Western medicines such as renin–angiotensin system (RAS) inhibitors are the mainstay of DKD treatment and have been successful in glycaemic management, hypertension control and risk reduction of disease onset and progression (National Kidney Foundation 2012). Beyond medications that control glycaemia and blood pressure, only treatment with RAS inhibitors has shown robust nephroprotective effect in randomized controlled trials (RCTs) (Anders et al. 2016). However, an unmet therapeutic need exists in DKD patients intolerant or unresponsive to current clinical drug treatment and patients presenting with a combination of deteriorating renal function and normo-albuminuria (Afkarian et al. 2016; Krolewski et al. 2017; Kramer et al. 2018; Zhang, Yang et al. 2019).

In recent years, an increasing number of published studies RCTs, case series and case reports have confirmed the efficacy of traditional Chinese medicine for DKD treatment. To facilitate the discovery of new therapeutic agents and strategies for patients with DKD, the screening of promising candidates from among natural products, including traditional Chinese medications used to alleviate symptoms associated with DKD, may offer insights into a more targeted approach for therapeutic development.

*Panax notoginseng* (Burk.) F.H. Chen (Araliaceae), also called Sanqi or Tianqi in China, is a highly valued and widely used herbal medicine in Asia. *Panax notoginseng* preparations (PNP) include Xuesaitong injection, Xueshuantong injection, Xuesaitong soft capsule, Xueshuantong capsules, Lulutong injection and *Panax notoginseng* saponin tablets. *P. notoginseng* has exhibited good therapeutic effects on the cardiovascular (Yang et al. 2014), cerebrovascular (Song et al. 2017) and nervous systems (Xie et al. 2018).

The therapeutic effects of PNP in DKD patients and their influence on renal function and albuminuria have not yet been systematically reviewed. Here, we evaluated the effects of PNP on DKD patients through a rigorous systematic review and meta-analysis of RCTs.

## Methods

### Search strategy

The databases PubMed, Embase, Medline (via Ovid SP), Cochrane Library, CINAHL (via EBSCO), China Biology Medicine disc (CBM disc), Wanfang, VIP and China National Knowledge Infrastructure (CNKI) were systematically searched from the date of their inception until May 2019. The following search terms were used: diabetic nephropathy, DKD and *Panax notoginseng* (Sanqi), *Panax notoginseng* saponins (Sanqizongzaogan), Lulutong, Xueshuantong, Xuesaitong. No language restriction was imposed. The reference lists of all retrieved articles were reviewed to identify additional articles missed using the above-mentioned search terms. The authors approved all the enrolled studies.

### Inclusion criteria

Studies meeting the following criteria were included: (1) study type: clinical RCTs using PNP as adjuvant treatment for DKD; (2) participants: the diagnostic criterion for DKD included adults with primary diabetes and persistent albuminuria/proteinuria; (3) interventions: RCTs comparing combined PNP therapy and conventional treatments versus conventional treatments alone were included (conventional treatments were antidiabetic agents and antihypertensive agents, etc.); (4) comparisons: as long as the conventional treatments were identical between the study groups in an RCT, the trial could be included; and (5) outcomes: albuminuria, proteinuria, serum creatinine (Scr), blood urea nitrogen (BUN), total cholesterol (TC), triglycerides (TGs), low-density lipoprotein cholesterol (LDL-C), high-density lipoprotein cholesterol (HDL-C) and fasting blood glucose (FBG).

### Exclusion criteria

The exclusion criteria were: (1) reviews, nonclinical studies and case observations; (2) non-RCTs, meta-analysis, case reports, editorials and meeting abstracts; (3) duplicated studies; (4) incorrect, incomplete, or not available studies data, or the study did not report at least one of the primary outcomes; (5) studies, in which control groups received an intervention that treatment groups did not receive; (6) combined with any herbal medicines in the experimental or control group during the treatment; and (7) improper outcome measures.

### Selection of studies and data extraction

A comprehensive search of the databases was performed by two researchers (Tang and Huang). The researchers deleted duplicate records, screened the titles and abstracts for relevance and identified them as excluded or requiring further assessment. We reviewed the full-text articles designated for inclusion and manually checked the references of the retrieved articles and previous reviews to identify additional eligible studies. Discrepancies were resolved by a discussion and reaching consensus. The following data were extracted from each study: first author, year of publication, number of patients, age, diabetes type, kidney function, course of disease, interventions, comparisons and outcomes, etc.

### Statistical analysis

Data were analysed using the Review Manager 5.3.0 statistical package (Cochrane Collaboration Software). For continuous variables, mean and SD were obtained from each study and pooled as mean difference (MD) (Duan et al. 2018).

If substantial heterogeneity occurred (*I*^2^ > 50% or *p* < 0.05), a random effects model was used to calculate the pooled MD (Chen et al. 2019). Publication/reporting biases were visually assessed using funnel plots. If no heterogeneity was observed, the fixed effects model was chosen. Subgroup analyses were performed to explore potential sources, whereby results were stratified by factors such as different conventional treatments, including PNP plus antihypertensive agents and PNP plus hypoglycaemic agents, etc.

## Results

### Study identification and selection

**Figure 1.**
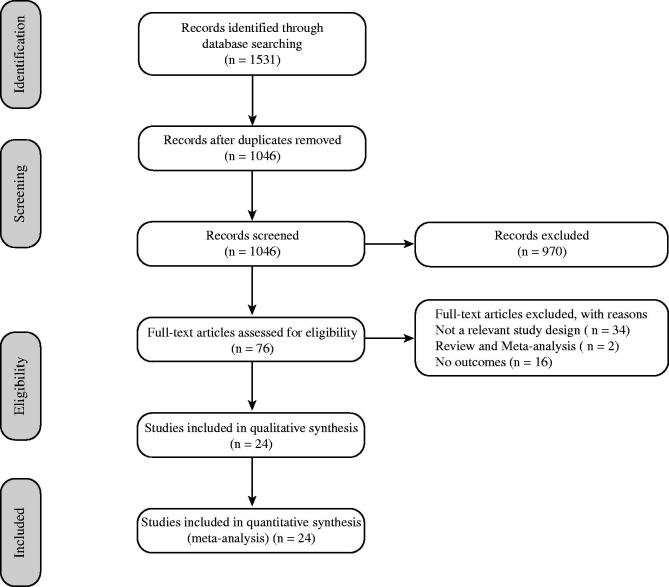
Selection process for the studies included in the meta-analysis.

In total, 1531 records were retrieved as a result of the initial database search. After the removal of 485 duplicate articles, 1046 records were eligible for further analysis. Based on the inclusion and exclusion criteria, 970 articles were excluded after reading their titles and abstracts. The remaining 76 full-text articles were assessed for eligibility. Studies with an irrelevant study design, non-RCTs, meta-analysis, studies that reported only combination treatment, and studies with combination specifics were excluded. Finally, a total of 24 RCTs were included in the meta-analysis (Jiang 2007; Kuang et al. 2008; Tao et al. 2008; Wang et al. 2008; Zhang and Wang 2008; Chen et al. 2011; Jia et al. 2011; Yang et al. 2011; Dai et al. 2012; Wang and Zeng 2012; Wu and Yan 2013; Xue et al. 2013; Yun et al. 2013; Li et al. 2014; Peng and Guo 2015; Zhang 2015; Wang 2015a, 2015b; Deng and Huang 2016; Li et al. 2016; Wang and Li 2017; Hu et al. 2018; Wang 2018; Zhang 2018). The selection process was conducted according to the Preferred Reporting Items for Systematic Reviews and Meta-analyses (PRISMA) guidelines and is shown in Figure 1.

### Study characteristics

The basic characteristics of the included studies are listed in Table 1. Twenty-four RCTs involving 1918 participants and published from 2002 until 2018 were included in the analysis. The number of participants in the studies ranged from 30 to 124. Among the 24 RCTs, 15 studies (Jiang 2007; Kuang et al. 2008; Tao et al. 2008; Zhang and Wang 2008; Chen et al. 2011; Yang et al. 2011; Dai et al. 2012; Wang and Zeng 2012; Wu and Yan 2013; Xue et al. 2013; Yun et al. 2013; Li et al. 2014; Peng and Guo 2015; Zhang 2015; Li et al. 2016) evaluated PNP plus angiotensin-converting enzyme inhibitors (ACEi) or angiotensin II receptor blockers (ARBs) compared with ACEi/ARB; six studies (Wang et al. 2008; Jia et al. 2011; Deng and Huang 2016; Wang and Li 2017; Wang 2018; Zhang 2018) assessed PNP plus antidiabetic agents compared with antidiabetic agents, and three studies (Wang 2015a, 2015b; Hu et al. 2018) evaluated PNP plus alprostadil compared with alprostadil.

**Table 1.** The characteristics of the included studies.

Study (year)	Sample size (M/F)	Age	Diabetes type	Kidney function	Course of disease	Intervention and control protocol		Method of administration	Duration	Reported outcomes
						Experimental	Control			
Chen et al. (2011)	72 (37/35)	T: 51.2 ± 2.8 C: 52.1 ± 3.1	2	NR	T: 3.3 ± 0.9 y C: 3.5 ± 0.8 y	PNP + CM (telmisartan)	CM (telmisartan)	P	1 m	Scr, BUN, TG
Dai et al. (2012)	80 (48/32)	T: 51.7 ± 5.2 C:52.2 ± 1.3	2	DKD stage III	T: 11.2 ± 2.2 y C: 11.3 ± 1.4 y	PNP + CM (benazepril)	CM (benazepril)	O	12 w	UAE, TC, TG, FBG
Deng and Huang (2016)	80 (43/37)	T: 49.51 ± 10.01 C: 51.55 ± 11.48	2	Albuminuria 30–300 mg/24 h	NR	PNP + CM (antidiabetic agents)	CM (antidiabetic agents)	P	3 w	UAE, Scr
Hu et al. (2018)	100 (37/63)	51.8 ± 10.1	2	NR	NR	PNP + CM (alprostadil)	CM (alprostadil)	P	2 w	24 h UP, TG, FBG
Jia et al. (2011)	30 (12/18)	48.5 ± 11.7	2	Proteinuria > 0.5 g/24 hours	8.51 ± 3.82 y	PNP + CM (antidiabetic agents)	CM (antidiabetic agents)	P	2 w	24 h UP, Scr, BUN, TG, LDL-C, HDL-C
Jiang (2007)	80 (44/36)	48–76	2	NR	5–18 m	PNP + CM (benazepril)	CM (benazepril)	O	1 m	24 h UP, Scr, BUN, TG, FBG
Kuang et al. (2008)	56 (30/26)	43–73	2	NR	6–21 y	PNP + CM (ramipril)	CM (ramipril)	O	30 d	UAE
Li et al. (2014)	100 (63/37)	T: 59.3 ± 6.4 C: 58.8 ± 5.9	2	Proteinuria < 0.5 g/24 hours	T: 11.4 ± 3.2 m C: 11.3 ± 2.8 m	PNP + CM (enalapril)	CM (enalapril)	O	4 w	BUN, TC, TG, LDL-C, HDL-C
Li et al. (2016)	60	NR	2	NR	NR	PNP + CM (valsartan)	CM (valsartan)	O	3 m	UAE, Scr, BUN, TG, LDL-C, HDL-C, FBG
Peng and Guo (2015)	124 (70/54)	T: 59.89 ± 8.24 C: 58.28 ± 8.50	2	Albuminuria 20–200 μg/min and GFR 70–130 mL/min or Proteinuria < 0.5 g/24 hours	T: 7.2 ± 3.1 y C: 7.5 ± 2.9 y	PNP + CM (valsartan)	CM (valsartan)	O	8 w	Scr, BUN, TC, TG
Tao et al. (2008)	80 (44/36)	48–76	2	NR	5–17 m	PNP + CM (irbesartan)	CM (irbesartan)	P	4 w	UAE, 24 h UP, Scr, BUN, TG, FBG
Wang and Li (2017)	78 (41/37)	T: 56.1 ± 7.1 C:54.5 ± 6.6	2	Albuminuria 20–200 μg/min or 30–300 mg/24 h	T: 6.5 ± 3.7 y C: 6.0 ± 2.7 y	PNP + CM (antidiabetic agents)	CM (antidiabetic agents)	O	12 w	UAE, TC, TG, LDL-C, FBG
Wang and Zeng (2012)	80 (35/45)	47–77	2	NR	4–17 m	PNP + CM (losartan)	CM (losartan)	O	24 w	24 h UP, Scr, BUN, TG, FBG
Wang (2015a)	43 (24/19)	T: 48.0 ± 11.0 C:51.0 ± 12.3	2	DKD stage III, albuminuria 20–200 μg/min or 30–300 mg/24 h	T: 6.8 ± 1.0 y C: 7.1 ± 0.9 y	PNP + CM (alprostadil)	CM (alprostadil)	P	2 w	UAE, Scr, BUN
Wang (2015b)	100 (57/43)	35–75	2	NR	5–22 y	PNP + CM (alprostadil)	CM (alprostadil)	P	4 w	24 h UP, Scr, FBG
Wang et al. (2008)	60	50.5 ± 10.5	2	Albuminuria 30–300 mg/24 h	6.5 ± 12.5 y	PNP + CM (antidiabetic agents)	CM (antidiabetic agents)	P	6 w	UAE, TC, TG
Wang (2018)	90 (61/29)	T: 64.4 ± 11.8 C: 63.5 ± 11.0	2	Albuminuria 30–300 mg/24 h	T: 2.0 ± 0.9 y C: 2.0 ± 1.0 y	PNP + CM (gliclazide and metformin)	CM (gliclazide and metformin)	P	3 w	FBG
Wu and Yan (2013)	120 (69/51)	T: 56.3 ± 5.2 C:52.2 ± 1.3	2	DKD stage III, albuminuria 20–200 μg/min or 30–300 mg/24 h	T: 10.4 ± 4.6 y C: 10.6 ± 5.2 y	PNP + CM (valsartan)	CM (valsartan)	O	24 w	TC, TG, FBG
Xue et al. (2013)	40	NR	2	DKD stage III	NR	PNP + CM (benazepril)	CM (benazepril)	P	2 w	UAE, Scr, BUN
Yang et al. (2011)	120 (69/51)	48–76	2	Albuminuria 30–300 mg/24 h	4–18 m	PNP + CM (losartan)	CM (losartan)	P	1 m	UAE, 24 h UP, HDL-C
Yun et al. (2013)	102 (61/41)	T: 53.5 ± 6.4 C:55.1 ± 7.2	2	Albuminuria 30–300 mg/24 h	T: 6.6 ± 3.1 y C:6.1 ± 2.7 y	PNP + CM (losartan)	CM (losartan)	O	12 w	UAE, TC, TG, LDL-C, FBG
Zhang and Wang (2008)	70 (39/31)	T: 46.22 ± 11.37 C:45.73 ± 10.33	2	Albuminuria 30–300 mg/24 h	6–11 y	PNP + CM (losartan)	CM (losartan)	P	4 w	UAE, TC, TG, LDL-C, HDL-C, FBG
Zhang (2018)	93 (54/39)	57.97 ± 5.11	2	DKD stage III	NR	PNP + CM (gliclazide and metformin)	CM (gliclazide and metformin)	O	3 m	UAE, Scr
Zhang (2015)	60	NR	2	DKD stage III, albuminuria 20–200 μg/min or 30–300 mg/24 h	>3 m	PNP + CM (valsartan)	CM (valsartan)	P	3 w	TC, TG, LDL-C, HDL-C

[Sample size] M/F: male versus female. [Age, Kidney function, Course of disease, Duration] T: tested group; C: control group; NR: not report; d: day; w: week; m: month; y: year. [Intervention and control protocol] CM: conventional medicine; PNP: Panax notoginseng preparation. [Reported outcomes] 24 h UP: 24 hours proteinuria; UAE: urinary albuminuria excretion; Scr: serum creatinine; BUN: blood urea nitrogen; TC: total cholesterol; TG: triglycerides; HLDL-C: high-density lipoprotein cholesterol; LDL-C: low-density lipoprotein cholesterol; FBG:fasting blood glucose. [method of administration] O: oral; P: parenteral.

### Risk of bias assessment

**Figure 2.**
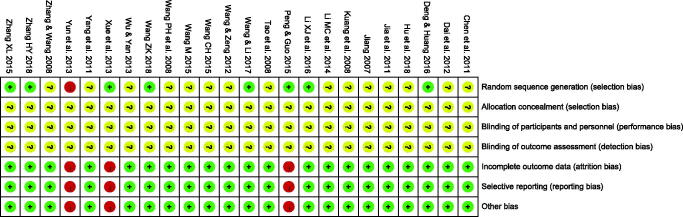
Risk of bias of included studies.

The risk of bias outcomes is summarized in Figure 2. Eight RCTs indicated that random numbers were used; in one trial (Yun et al. 2013), a sequence was generated based on hospital or clinic record numbers. The remaining studies did not describe the randomization method in detail. All the included trials did not describe the concealment and blinding method. Three RCTs (Xue et al. 2013; Yun et al. 2013; Peng and Guo 2015) reported case shedding.

### Efficacy of PNP

As the use of different conventional medicines (CM) such as RAS inhibitors may affect the primary outcomes, the studies were separated into three groups prior to the meta-analysis, according to the trial application of the CM in each arm. Notably, the conventional concurrent DKD treatments recommended by the guidelines were applied equally in both study arm in all included studies. The three groups were: (1) PNP plus ACEi/ARB versus ACEi/ARB; (2) PNP plus antidiabetic agents versus antidiabetic agents; and (3) PNP plus alprostadil versus alprostadil.

### Effect on kidney function

#### Albuminuria

**Figure 3.**
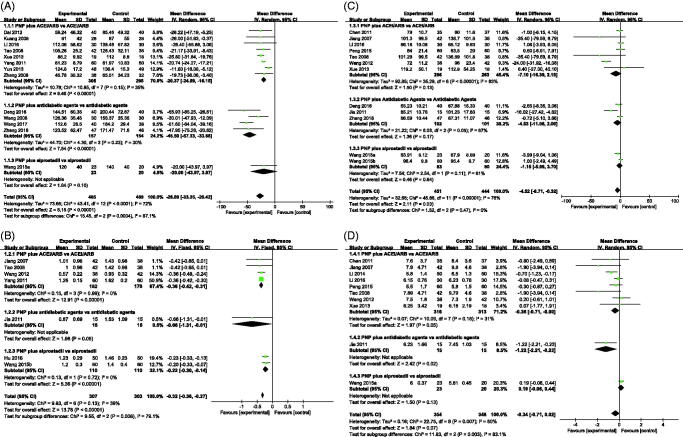
Forest plot of kidney function outcomes for: (A) albuminuria; (B) proteinuria; (C) serum creatinine; (D) blood urea nitrogen. ACEi: angiotensin-converting enzyme inhibitors; ARBs: angiotensin receptor blockers; PNP: *Panax notoginseng* preparations.

Thirteen studies with 954 participants reported data on urinary albumin excretion at the end of treatment. Treatment with PNP plus CM apparently lowered the levels of albuminuria compared to the CM group; the difference was statistically significant (MD –26.89 mg, 95% CI: –33.35 to –20.42; *p* < 0.001, Figure 3(A)) with heterogeneity (*I*^2^ = 72%). Subgroup analysis suggested that different CM could be the sources of heterogeneity. When we compared PNP plus ACEi/ARB with ACEi/ARB, a slightly lower albuminuria level was observed at the end of treatment (MD –20.37 mg, 95% CI: –24.59 to –16.15; *p* < 0.001, Figure 3(A)) with no heterogeneity (*I*^2^ = 35%). Similar results were observed for PNP plus antidiabetic agents versus antidiabetic agents (MD –45.50 mg, 95% CI: –57.33 to –33.68; *p* < 0.001, *I*^2^ = 30%, Figure 3(A)).

#### Proteinuria

Seven studies including 610 patients measured 24 h proteinuria at the end of treatment. Compared with CM, PNP plus antidiabetic agents apparently reduce the proteinuria without heterogeneity (MD –0.32 g/24 h, 95% CI: –0.36 to –0.27; *p* < 0.001, *I*^2^ = 39%, Figure 3(B)). Similar results were observed in a subgroup analysis including studies based on different CM (Figure 3(B)).

#### Serum creatinine

Scr levels at the end of treatment were evaluated in 896 patients from 12 studies. Pooled estimation of the included trials showed that adjuvant PNP may reduce the Scr levels compared with CM, with heterogeneity (MD –4.52 μmol/L, 95% CI: –8.71 to –0.32; *p* < 0.001, *I*^2^ = 76%, Figure 3(C)), and random model was used. Subgroup analysis including studies based on different CM treatments showed similar results (Figure 3(C)). Sources of heterogeneity were not identified; low-quality evidence suggested that PNP may contribute to the end of treatment Scr differences.

#### Blood urea nitrogen

BUN levels at the end of treatment were evaluated in 702 patients from 10 studies. The pooled estimated effect showed that BUN levels in the PNP plus CM group did not differ from those in the CM group, heterogeneity was present (MD –0.34 mmol/L, 95% CI: –0.71 to 0.02; *p* =  0.07, *I*^2^ = 60%, Figure 3(D)). Subgroup analysis revealed that different CM may have been the source of heterogeneity.

### Secondary outcomes

#### Lipid

**Figure 4.**
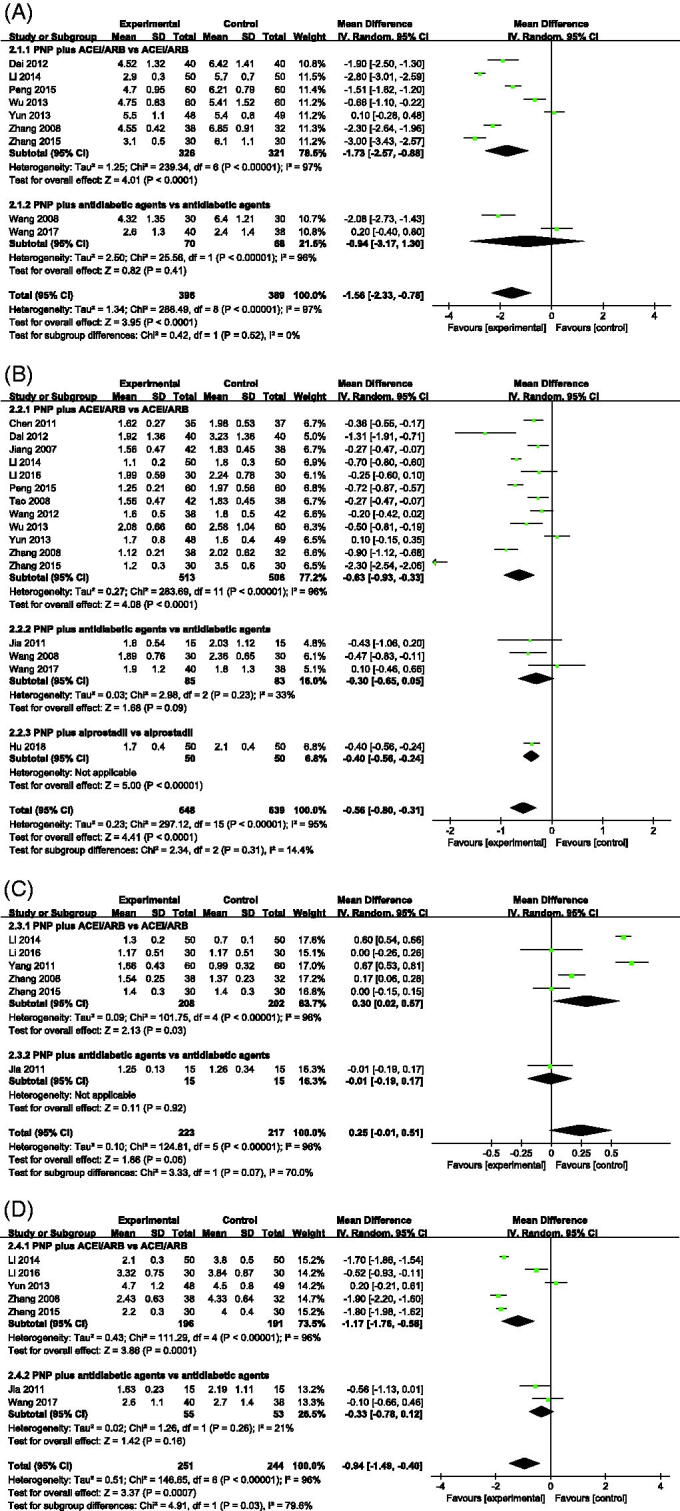
Forest plot of lipid outcomes for: (A) TC; (B) TG; (C) HDL-C; (D) LDL-C. ACEi: angiotensin-converting enzyme inhibitors; ARBs: angiotensin receptor blockers; PNP: *Panax notoginseng* preparations.

The TC concentrations at the end of treatment were evaluated in 785 patients from nine studies. The pooled estimated effect showed that the TC levels were significantly different in the PNP plus CM groups, compared with the CM group, with heterogeneity (MD –1.56 mmol/L, 95% CI: –2.33 to –0.78; *p* < 0.001, *I*^2^ = 97% Figure 4(A)), and random model was used. Subgroup analysis showed similar results; sources of heterogeneity were not identified.

The TG concentrations at the end of the treatment were evaluated in 1287 patients from 16 studies. TG levels differed significantly between the CM and PNP plus CM groups with heterogeneity (MD –0.56 mmol/L, 95% CI: –0.80 to –0.31; *p* < 0.001, *I*^2^ = 95%, Figure 4(B)), and random model was used. Similar results were observed in a subgroup analysis by the type of CM.

HDL-C concentrations at the end of treatment were evaluated in 440 patients from six studies. PNP plus ACEi/ARB apparently increased HDL-C levels compared with ACEi/ARB with heterogeneity (MD 0.30 mmol/L, 95% CI: 0.02–0.57; *p* < 0.001, *I*^2^ = 96%, Figure 4(C)), and random model was used. No significant differences in HDL-C concentrations were observed between PNP plus antidiabetic agents and antidiabetic agents.

Seven trials mentioned LDL-C concentrations. PNP plus ACEi/ARB was more effective in reducing the LDL-C content. The difference between the two groups was statistically significant (MD –0.94 mmol/L, 95% CI: –1.49 to –0.40; *p* < 0.001, *I*^2^ = 96%, Figure 4(D)); heterogeneity was present, and random model was used. However, the results showed no significant differences between PNP plus antidiabetic agents versus antidiabetic agents.

#### Fasting blood glucose

**Figure 5.**
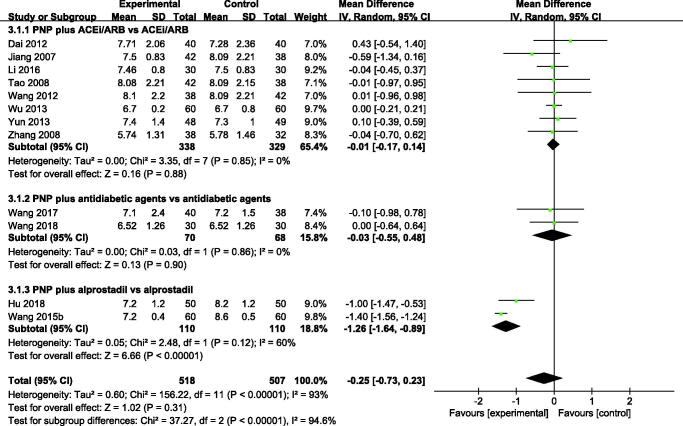
Forest plot of FBG. ACEi: angiotensin-converting enzyme inhibitors; ARBs: angiotensin receptor blockers; PNP: *Panax notoginseng* preparations.

Twelve trials including 1025 patients mentioned FBG. No significant differences in FBG reduction were observed between PNP plus ACEi/ARB or antidiabetic agents and ACEi/ARB or antidiabetic agents. However, PNP plus alprostadil was more effective in FBG reduction (MD –1.26 mmol/L, 95% CI: –1.64 to –0.89; *p* < 0.001, Figure 5); the heterogeneity among the studies was large (*I*^2^ = 60%).

## Discussion

Recently, an increasing number of studies, including RCTs, case series and case reports, have indicated the efficacy of traditional Chinese medicine for DKD treatment, PNP which contain active herbal ingredients, have attracted high research interest. However, the efficacy of PNP as adjuvant therapy for DKD has not yet been demonstrated. In this study, adequate RCTs were included after a comprehensive search, and subgroup analysis was performed. Overall, 24 RCTs involving 1918 participants were included to evaluate the efficacy of PNP in addition to conventional DKD therapies.

As an adjunct treatment, PNP may decrease the albuminuria, proteinuria, and Scr, TC, TG, and LDL-C levels in DKD patients, compared with the CM only group. Furthermore, PNP may increase the levels of HDL-C when combined with some CM. This study demonstrated that PNP may be applied as an adjunct treatment for DKD to achieve better renal outcomes without heterogeneity. PNP combined with antidiabetic agents caused moderate short-term reduction of the albuminuria or proteinuria in comparison with antidiabetic agents only. In combination with ACEi/ARB or alprostadil, the lowering albuminuria effect of PNP was mild to moderate from a clinical perspective. Considering the failure of dual RAS inhibitor therapy, PNP could be a potential choice for DKD patients treated with ACEi/ARB to achieve greater albuminuria reduction within a short time.

The renal protective effect of PNP in these RCTs may be related to particular bioactive compounds contained in the herbal ingredients included. The main bioactive ingredients of *P. notoginseng* consist of ginsenoside Rg1, ginsenoside Rb1 and notoginsenoside R1 (Song et al. 2017). Ginsenoside Rg1 may potentially protect against diabetic nephropathy via reduction of oxidative stress and inhibition of the TGF-β1/Smads signalling cascade (Du et al. 2018). Ginsenoside Rb1 may represent an antioxidant-based approach to slow the progression of chronic kidney disease (CKD) at the early stages (Xu et al. 2017). Notoginsenoside R1 exerts nephroprotective effects against diabetic nephropathy through the inhibition of apoptosis and renal fibrosis caused by oxidative stress (Zhang, Zhang et al. 2019).

Another valuable finding of our systematic review was the lipid-lowering influence of PNP. As a complementary medicine, PNP could strengthen the beneficial effects on lipids by decreasing the levels of TC, TG and LDL. Furthermore, PNP combination treatment may increase HDL levels. Dyslipidaemia, especially including elevated TG and decreased HDL levels, is an important risk factor that contributes to the development of CKD in patients with diabetes (Thomas et al. 2015; Zac-Varghese and Winocour 2018).

Moreover, none of the included trials mentioned adverse events. The safety of PNP is likely one of the critical reasons for their popularity. However, it should not be assumed that PNP are absolutely safe, just because they are natural products. Well conducted studies are urgently needed to evaluation the safety of PNP for DKD treatment.

This study has several limitations. First, the quality of the included RCTs was generally low according to Cochrane’s risks of bias tool. Thus, we have to suspect the facticity of these included RCTs. The lack of high-quality studies impedes the generalization of the data and the definitive assessment of their relevance. Second, the diagnostic criteria for the evaluation of efficacy were not uniform among the studies. The diagnosis standard of eight trials included unclear DKD diagnostic criteria. Third, our analysis was based on 24 RCTs, and most of them had a relatively small sample size (*n* < 100). In addition, all 24 included trials were conducted in China, furthermore, all articles were written in Chinese, no English articles were included, and no negative studies were generated. Thus, the effect of PNP reported in this review may not be generalizable to other populations. Last but not least, due to the short follow-up periods and small numbers of clinical events in terms of mortality and progression to ESKD, the long-term benefit of PNP is yet to be determined. None of the included trials mentioned adverse events; thus, well-conducted RCTs are urgently needed to evaluate the safety of PNP.

## Conclusions

To the best of our knowledge, this is the first meta-analysis to investigate the efficacy of PNP as an adjuvant treatment for DKD. Overall, the use of adjuvant PNP in combination with CM showed promise for the improvement of renal function, such as reduction of albuminuria, proteinuria and Scr levels and optimization of the metabolism of serum lipids, such as reduction of TC, TG and LDL-C in patients with DKD. However, due to the quality of the original studies, more well-conducted, multicentre, large sample, high quality RCTs are needed to provide high-quality evidence.
